# Diagnostic Value of the Texture Analysis Parameters of Retroperitoneal Residual Masses on Computed Tomographic Scan after Chemotherapy in Non-Seminomatous Germ Cell Tumors

**DOI:** 10.3390/cancers15112997

**Published:** 2023-05-31

**Authors:** Clémence Fournier, Clémence Leguillette, Eric Leblanc, Marie-Cécile Le Deley, Aurélien Carnot, David Pasquier, Alexandre Escande, Sophie Taieb, Luc Ceugnart, Loïc Lebellec

**Affiliations:** 1 Department of Medical Oncology, Centre Hospitalier de Roubaix, 59100 Roubaix, France; clemence.fournier@ch-roubaix.fr; 2 Clinical Research Department, Centre Oscar Lambret, 59000 Lille, France; c-leguillette@o-lambret.fr (C.L.); m-ledeley@o-lambret.fr (M.-C.L.D.); 3 Department of Surgical Oncology, Centre Oscar Lambret, 59000 Lille, France; e-leblanc@o-lambret.fr; 4 Department of Medical Oncology, Centre Oscar Lambret, 59000 Lille, France; a-carnot@o-lambret.fr; 5 Academic Department of Radiation Oncology, Centre Oscar Lambret, 59000 Lille, France; d-pasquier@o-lambret.fr; 6 Univ. Lille, CNRS, Centrale Lille, UMR 9189–CRIStAL, 59000 Lille, France; aescande@clinique-psv.fr; 7 Department of Radiotherapy, Clinique Léonard de Vinci, 59187 Dechy, France; l-ceugnart@o-lambret.fr; 8 Department of Radiology, Centre Oscar Lambret, 59000 Lille, France; s-taieb@o-lambret.fr

**Keywords:** non-seminomatous germ cell tumor, surgery, residual lesions, radiomics, score, prediction

## Abstract

**Simple Summary:**

Approximately 50% of residual masses requiring surgery after chemotherapy in non-seminomatous germ cell tumors consist of necrosis/fibrosis. We aimed to develop a score to predict the malignant character of these masses to avoid surgical overtreatment and the complications. In a retrospective study, we included 76 patients, with 149 residual masses. The score was constructed from the textures of the masses on a computed tomography scan with a free software. In conclusion, our score may help in the prediction of the malignant nature. However, these results are insufficient to simply select patients for surgery.

**Abstract:**

After chemotherapy, patients with non-seminomatous germ cell tumors (NSGCTs) with residual masses >1 cm on computed tomography (CT) undergo surgery. However, in approximately 50% of cases, these masses only consist of necrosis/fibrosis. We aimed to develop a radiomics score to predict the malignant character of residual masses to avoid surgical overtreatment. Patients with NSGCTs who underwent surgery for residual masses between September 2007 and July 2020 were retrospectively identified from a unicenter database. Residual masses were delineated on post-chemotherapy contrast-enhanced CT scans. Tumor textures were obtained using the free software LifeX. We constructed a radiomics score using a penalized logistic regression model in a training dataset, and evaluated its performance on a test dataset. We included 76 patients, with 149 residual masses; 97 masses were malignant (65%). In the training dataset (*n* = 99 residual masses), the best model (ELASTIC-NET) led to a radiomics score based on eight texture features. In the test dataset, the area under the curve (AUC), sensibility, and specificity of this model were respectively estimated at 0.82 (95%CI, 0.69–0.95), 90.6% (75.0–98.0), and 61.1% (35.7–82.7). Our radiomics score may help in the prediction of the malignant nature of residual post-chemotherapy masses in NSGCTs before surgery, and thus limit overtreatment. However, these results are insufficient to simply select patients for surgery.

## 1. Introduction

Testicular cancer is the most common malignancy among men aged between 20 and 35 years [1]. Malignant tumors of the testis are found in 98% of germ cell tumors (GCTs) and can be divided into two groups: pure seminomatous germ cell tumors, which are more common, and non-seminomatous germ cell tumors (NSGCTs) in 45% of cases. NSGCTs consist of embryonal carcinoma, teratoma, yolk sac tumor, choriocarcinoma, and seminoma, in varying proportions [2]. At the time of diagnosis, approximately 60–70% of patients have stage I disease (localized disease), and 30–40% of patients are diagnosed with metastatic disease. According to the International Germ Cell Cancer Collaborative Group (IGCCCG) risk classification, patients with metastatic disease are treated with three or four cycles of platin-based polychemotherapy protocols [3], eventually followed by residual tumor resection according to the size and an objective response after chemotherapy [4,5]. 

Residual lesions should be evaluated using thoraco-abdomino-pelvic computed tomography. For NSGCTs, any residual mass greater than 1 cm in the minor axis on post-chemotherapy re-evaluation computed tomography (CT) should be surgically removed [6]. Indeed, unlike pure seminomatous germ cell tumors, the use of 18-fluorodeoxyglucose positron emission tomography (18-FDG-PET) to select NSGCTs with a residual mass for surgery is not relevant because of false-negative teratomas [7]; the only selection criterion is therefore the size criterion on the CT scan. Histological analysis of these residual masses revealed 50% necrosis/fibrosis, 40% teratomas, and 10% active cancer cells [8,9,10,11]. 

Residual tumor resection does not solely consist of tumor removal but also regional surgery [12], with combined procedures in 13.5% of cases with inherent risks (vascular, bone, and visceral surgery) [13,14,15]. 

Several studies have attempted to determine predictive factors to classify residual masses, including pre- and post-chemotherapy tumor marker assays, initial presence of teratoma, residual mass size, and regression of masses during chemotherapy [16,17,18,19]. Two models have shown potential in predicting the histology of residual masses: Vergouwe’s model, based on six predictive factors (histology of the primary tumor, serum alpha-fetoprotein (AFP) levels before chemotherapy, human chorionic gonadotrophin (hCG), lactate dehydrogenase (LDH), residual mass size measured by CT, and variation in mass size) [20]; and Leão’s model (Princess Margaret model), based on four predictive factors (presence of teratoma in the orchidectomy, pre-chemotherapy AFP, size of masses before chemotherapy, and variation in mass size during chemotherapy) [21]. However, the discrimination ability of both models to distinguish non-malignant/malignant residual lesions is not sufficient to safely select patients for resection and thus prevent overtreatment [22]. Thus, to date, no standard imaging procedure (including PET) or prognostic model has reliably predicted the histology of the residual mass.

Recently, a field of research known as radiomics has been developed. It involves identifying associations between qualitative and quantitative information extracted from standard imaging to identify predictive and/or prognostic information/factors to facilitate medical decision making [23,24,25]. This analysis of different radiomics parameters, generally grouped in a predictive or prognostic algorithm, aims to improve the purely visual analysis performed by radiologists. 

Several studies have suggested the predictive and/or prognostic value of radiomics (sometimes coupled with clinical data) in oncology, particularly on lymph node status in colorectal, lung, esophageal, and bladder cancers [26,27,28,29,30].

Radiomics in residual masses after chemotherapy in NSGCTs, possibly coupled with clinicopathological data, has been studied with promising results [31,32]. However, these models remain poorly described or consist of machine-learning methods that limit their use in daily practice and the performance of these methods has remained insufficient to change practices.

The present study aimed to develop and validate a predictive model including a radiomics score based on post-chemotherapy contrast-enhanced CT texture analysis for the histopathological presence of malignant versus benign residual retroperitoneal masses in patients with NSGCTs.

## 2. Materials and Methods

### 2.1. Patients

In this retrospective monocentric study, all consecutive patients with histologically proven NSGCTs, between September 2007 and July 2020 at the Oscar Lambret Center, were screened. Inclusion criteria were a primary testicular location and retroperitoneal lymph node involvement, patients who received platinum-based chemotherapy, and patients who underwent retroperitoneal lymphadenectomy after normalization of tumor markers as recommended. Participants were identified from a hospital database of patients with a germ cell tumor who underwent surgery for residual masses after chemotherapy. Exclusion criteria were patients for whom the pathological results of orchidectomy were not available, those with a primary extra-testicular location, and those who had not received platinum-based chemotherapy. Additionally, we excluded patients with post-chemotherapy injected CT scans unavailable or deemed to be of poor quality for defining residual masses, and those who refused to participate in the study.

Clinical, scanographic, and pathological data were retrospectively extracted from hospital medical records. Data were collected for each patient and residual mass. 

The Institutional Review Board of the Oscar Lambret Center has confirmed that no ethical approval was required (number CEC-2023-006). This study was conducted in compliance with the “reference methodology” (MR004) adopted by the French Data Protection Authority (Commission nationale de l’informatique et des libertés; CNIL). Informed consent was waived and we checked that patients did not object to the use of their clinical data for research purposes. All data were pseudonymized. 

### 2.2. Texture Analysis

Tumor texture analysis was performed on contrast-enhanced CT images acquired during the portal phase. After loading the DICOM (digital imaging and communications in medicine) anonymized files, each retroperitoneal residual mass with an available histopathological correlation was delineated in 3D using LifeX (version 7.2.0, www.lifexsoft.org), free software for calculating radiomics features in multimodal imaging. Trained radiologists reviewed and corrected all delineations. Residual masses were labeled as either “benign” (necrosis or fibrosis) or “malignant” (viable tumor or teratoma) on histopathology. Residual masses with inconclusive correlations between CT imaging and histopathology were excluded from further analysis.

Before feature extraction, all images underwent standardized preprocessing as follows: distance from neighbors of 1; spatial resampling to 2 × 2 × 2 mm; intensity discretization to a bin width of 10; and absolute intensity rescaling using a scale from −1000 to 3000 [33]. 

### 2.3. Statistical Analysis

We split the sample of residual lesions into two datasets: a training dataset (including 2/3 of the entire sample) to build the model and a test dataset (including 1/3 of the sample) to evaluate the model performance.

We modeled the risk of residual malignant lesions using logistic regression, with the explanatory variables as continuous variables leading to the construction of a score. It was not possible to calculate the number of lesions required for this type of modeling, given the unknown parameters studied and the modeling chosen (penalization by ELASTIC-NET or another approach). However, we could ensure a certain power by considering a simplified approach consisting of reducing our model to the analysis of the association between the risk of residual malignant lesions and a binarized factor: high versus low score. Overall, the expected proportion of benign lesions is 50%. For a binary factor (R+/R−) equally distributed in the population (pR+ = pR− = 50%), a total of 110 lesions were necessary to ensure a power of 90% if this factor was associated with an OR of 5.4, such that the probability of a benign lesion was 70% if R− and 30% if R+ (or the reverse). The test was performed at the two-sided 0.001% threshold, to account for the multiplicity of the tests. Assuming that a patient would have an average of 1.5 lesions, 74 patients were required in the training sample to analyze 110 lesions. The test sample had to include 65 lesions. Therefore, 110 patients were required to ensure the analysis of 165 residual masses. Assuming an annual recruitment of approximately 15 patients at the Oscar Lambret Center, the analysis of patients over a period of 7.5 years (2013–2020) should allow for this number of patients to be reached. 

The classical methods of descriptive statistics between the two groups (“benign” and “malignant”) were used. The residual masses were separated into two samples: a training sample (2/3) named “train” and a test sample (1/3) named “test”. A predictive model to characterize the malignant or benign nature of the residual masses was developed on the training sample and then tested on the test sample. 

Considering high-dimensional data, we first kept the relevant variables (removing extraction-specific features alone, those with more than 50% missing data, those identical for all lesions, and those not conforming to feature definitions described by the Imaging Biomarker Standardization Initiative (IBSI) [34]). Then, to make the distribution of feature values comparable and of the same order, the variables were centered and reduced. Second, the correlations between the remaining variables were analyzed. The Spearman correlation coefficient was calculated for each feature in each pair. We then ordered the coefficients in descending order. If the correlation was greater than strictly 0.9 (or <−0.9), we performed a Wilcoxon Mann Whitney test for the two correlated factors, to test the association between the distribution of each feature and the variable “benign” vs. “malignant” lesion. The feature obtaining the test with the lowest *p*-value was retained in the analysis [35]. Finally, the selection of radiomics features associated with the malignant character of the histology of the residual masses was performed using penalized logistic regression in the training set [36]. We fitted two models: ELASTIC-NET and least absolute shrinkage and selection operator (LASSO). For each of the models, a grid search approach using a five-fold cross-validation provided the penalty term(s) (one term for LASSO and two for ELASTIC-NET) that minimizes the average deviance on the different folds. We then selected the model with the best AUC on the training set. Based on the selected model, we calculated a radiomics score that corresponds to the probability of a lesion to be malignant, derived from the linear combination of the selected features, weighted by their regression coefficients. We estimated the sensitivity, specificity, positive and negative predictive values, and their 95% exact binomial confidence intervals (95%CI) considering several thresholds to classify the predictions as benign versus malignant.

The performance of the radiomics score built on the training dataset was finally evaluated on the test dataset.

The impact of adding clinico-biological factors to the radiomics score on the discrimination ability was also analyzed within the limits of the available data by comparing the AUC of the two models [37].

These analyses were performed by residual mass.

Stata v17.0 software (StataCorp. 2009. Stata statistical software: Release 11. College Station, TX, USA, StataCorp LP) was used for the analyses. The reported statistical significance levels were all two-sided, with the statistical significance set at 0.05.

## 3. Results

### 3.1. Patient and Residual Masses Characteristics 

**Figure 1:**
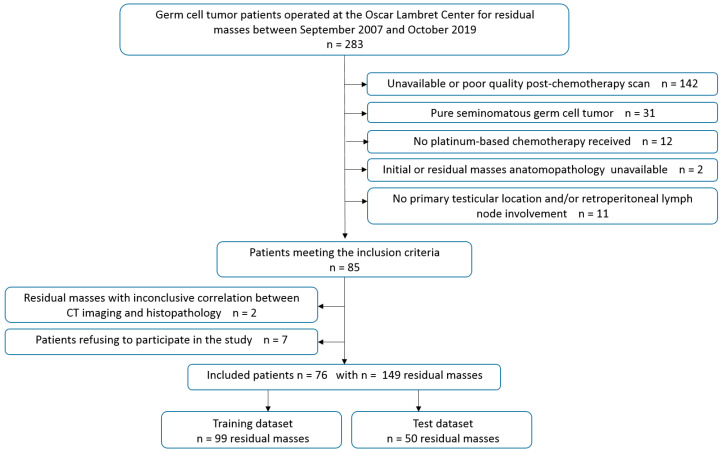
Flowchart of patients included in the study.

Of 283 patients with a germ cell tumor who underwent surgery for residual masses, 76 were finally included in the study (Figure 1). 

The patient and tumor characteristics are presented in Table 1. The median age of the patients was 28 years (range, 17–46 years). The initial pathology of orchidectomy specimens described teratomas in 28.9% of the patients. All patients received cisplatin-etoposide-bleomycin (PEB) and at least three cycles of chemotherapy. Some patients (*n* = 7, 9.2%) received second-line chemotherapy with vinblastine-ifosfamide-cisplatin (*n* = 5) or participated in a clinical trial (*n* = 2). CT was performed at a median time of 4.5 (range, 0.3–365) weeks after chemotherapy, and lymphadenectomy was performed at a median time of 6.7 (range, 0.0–40) weeks after the CT scan. Most patients presented with one residual mass (*n* = 45, 59.2%) or two to five (*n* = 28, 36.8%). The presence of a teratoma at diagnosis was significantly associated with the risk of a malignant residual mass (*p* = 0.02). 

The overall sample (*n* = 149 residual masses) was split between the training dataset, which included 99 residual masses (*n* = 65 malignant), and the test dataset, which included 50 residual masses (*n* = 32 malignant). Descriptions of these two datasets are presented in Appendix A, and the pathological results of the residual masses are summarized in Table 2.

### 3.2. Radiomics Feature Selection and Radiomics Score Construction

We selected a total of 105 relevant radiomics features from 178 radiomics features extracted per lymph node. 

Based on the analysis of their correlations (illustrated in Appendix B), we reduced the number of radiomics features to 32.

Finally, based on the training dataset, we identified as the best model a regularized ELASTIC-NET logistic regression (with alpha = 0.5 and lambda = 0.8), including a combination of eight factors from the thirty-two remaining texture features. The formula for the resulting radiomics score is detailed in Appendix C. A good performance was observed in the training dataset with an AUC of 0.856 (95%CI, 0.772–0.939).

### 3.3. Choice of Probability Threshold to Maximize Sensitivity

To define the probability threshold classifying the predictions as benign versus malignant, we first selected the value that maximized sensitivity while ensuring a specificity greater than 65%. If the probability calculated by the model was greater than or equal to the threshold, the lesion was classified as malignant by the model. The chosen threshold resulted in a misclassification for 7 of the 65 malignant residual masses (classified as benign) and for 11 of the 34 benign residual masses (classified as malignant). The model performance is summarized in Table 3. 

Other thresholds were then explored. Using the threshold that maximized the Youden index, the best compromise between sensitivity and specificity, we determined a sensitivity of 86.2% (75.3–93.5) and a specificity of 76.5% (58.8–89.3), with nine residual malignant masses classified as benign and eight residual benign masses as malignant. In contrast, when using the threshold that maximized a weighted mean of sensitivity and specificity giving twice the weight to sensitivity, only five residual malignant masses were classified as benign and twelve residual benign masses as malignant. 

### 3.4. Performance of the Radiomics-Score on the Test Data Dataset

**Figure 2:**
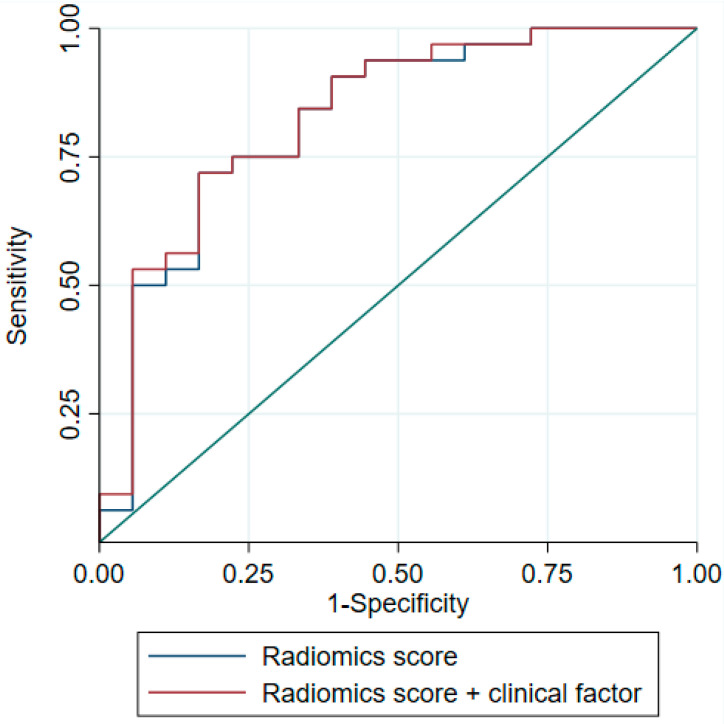
ROC curves of the models on the test base. ROC curve: receiver operating characteristic curve. The blue line represents the ROC curve of the model including the radiomics score defined by ELASTIC-NET logistic regression, estimated on the test dataset. The red line represents the ROC curve of the model including the radiomics score defined by ELASTIC-NET logistic regression plus the presence of teratoma in the orchidectomy, estimated on the test dataset.

The model was applied to the test sample containing 50 residual masses (18 benign and 32 malignant). As illustrated by Figure 2, the AUC in the test dataset was good at 0.823 (95%CI, 0.693–0.953), significantly higher than 0.50. 

Estimates of sensitivity, specificity, and positive and negative predictive values are detailed in Table 3, considering the three thresholds defined on the training dataset. Using the first threshold, we found a false-negative classification of three malignant residual masses and a false-positive classification of seven benign residual masses. Using the second threshold that maximized the Youden index, we found a false-negative classification of five malignant residual masses and a false-positive classification of six benign residual masses. When using the third threshold, which maximized the weighted mean giving twice the weight to sensitivity, we found a false-negative classification of two malignant residual masses and a false-positive classification of ten benign residual masses. Consequently, we observed that the sensitivity could be maintained in the test dataset, while the specificity largely decreased. 

### 3.5. Distribution of the Radiomics Score According to Clinical Factors

**Figure 3:**
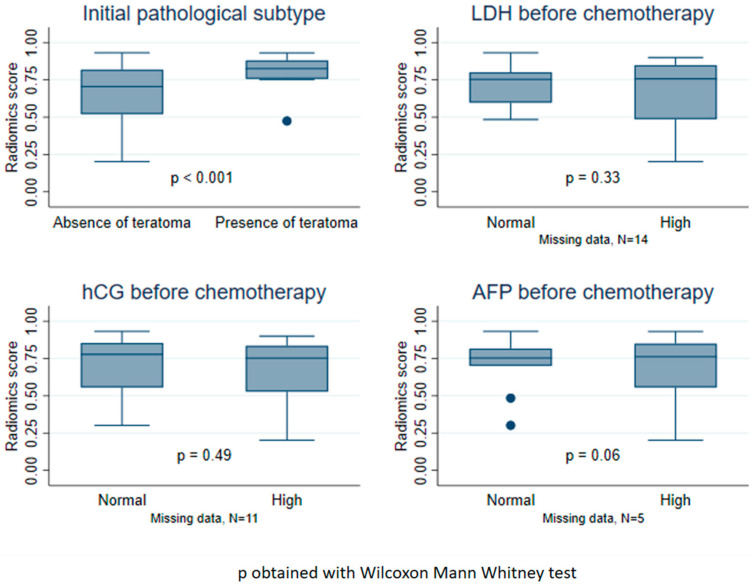
Boxplots of the radiomics score according to clinical factors, on the test dataset. LDH = Lactate dehydrogenase level before chemotherapy. hCG = Human chorionic gonadotrophin level before chemotherapy. AFP = Alpha-fetoprotein level before chemotherapy.

As illustrated by Figure 3, we observed a strong association between the presence of teratoma in the orchidectomy and the radiomics score (*p* < 0.001). We did not observe any significant association between the other clinical characteristics (LDH, hCG, and AFP levels before chemotherapy) and the radiomics score.

### 3.6. Evaluation of the Value of Adding Clinical Factors to the Radiomics Score

To improve our model, we included the clinico-biological variables validated in other studies [20]. Unfortunately, we decided to only consider the presence of teratoma in the orchidectomy specimen, as the other variables had too many missing data. However, this addition did not improve the performance of the model; in the test sample, as illustrated by Figure 2, AUC was estimated at 0.830 (95%CI, 0.704–0.956), not significantly different from the model without the clinical factor (*p* = 0.13, AUC equality test).

## 4. Discussion

The objective of our study was to establish a radiomics score from a training dataset to distinguish the benign from malignant nature of residual post-chemotherapy masses in NSGCTs, and to evaluate its performance in a test sample. It is the first study performed in France on radiomics in the classification of residual masses post-chemotherapy in NSGCTs aiming at limiting the overtreatment of patients in whom only necrosis remains.

The radiomics score model developed in our study achieved a good performance with an AUC of 0.823 (0.693–0.953), sensitivity of 90.6% (75.0–98.0), and specificity of 61.1% (35.7–82.7) in the test sample.

The addition of the validated clinical variable available in our study (presence of teratoma during orchidectomy) did not significantly improve the discriminative ability of our score. 

Our study has several strengths: the entirety of each mass was delineated, which allows a global consideration of its texture; all delineations were reviewed by experienced radiologists; the methods used to extract features and to calculate the radiomics score are clearly stated, contrary to the two previous studies; and the discriminative capacity of the score was evaluated on a validation test sample.

The results of our study are similar to those of the two previous studies on the subject in terms of patient characteristics and discriminative capacity [31,32]. Lewin et al. analyzed 102 residual masses from 77 patients in a retrospective study; their radiomics signature was associated with an AUC of 0.74 (+/−0.028), without splitting their population and validation in a test sample, in contrast to our study [31]. Baessler et al. used machine-learning methods to predict the histology of 204 residual masses from 80 patients. They also split their population into two samples and obtained in the validation test sample an AUC of 0.81, sensitivity of 84%, and specificity of 78%. Contrary to our study, the method used by Baessler et al. is not described enough to be reproduced in daily practice [32]. Our results are also consistent with those of other studies evaluating the discriminative ability of radiomics scores for lymph node status in other cancers, such as lung, colon, and esophageal squamous cell carcinomas (between 76 and 84%) [26,27,28,29,38].

However, our study has some limitations. This was a retrospective study, and the required number of participants was not reached. However, we included 76 patients with a total of 149 residual masses (about 2 lesions per patient). Larger studies with independent test datasets are required to increase the external validity. In our sample, we observed 38% benign lesions, lower than that reported in other studies. In addition, the data analyzed are not the raw data. They are extracted from different tomography machines; the thickness of the slides and voxel size may be different according to exams. Harmonization of the data could have been achieved, and recommendations for good practices were implemented but without a consensus on such processes [39,40]. However, to generalize the results, we standardized the image preprocessing [33] and only retained features that conformed with the definitions of IBSI features [34]. In our analyses, we modeled the risk of residual malignant lesions assuming that multiple lesions in a single patient were independent, which can be deemed too simplistic. Lastly, we acknowledge the fact that some other approaches could have been considered, such as more complex machine-learning tools. The interest of the method used in our study is to lead to a predictor that can be easily used by others. 

We know that evolution is different between mature and immature teratoma, and it will be interesting to dissociate these two entities. However, this precision was not available in most of histopathological results and both subtypes can be mixed.

We chose to find a threshold that favors sensitivity while maintaining a high specificity, but we could also have chosen to maximize sensitivity at the expense of specificity to maximize the chances of diagnosing malignant residual masses. However, by decreasing specificity, we move away from the goal of selecting patients for surgery. Finally, there were too few clinico-biological factors available in our present study to be correctly evaluated. Indeed, the patients in our study were operated on at Oscar Lambret Cancer Center (a reference center for this type of surgery), but their initial management had often been performed in other centers, and, thus, the radio-clinico-biological data prior to surgery (in particular, the pre-chemotherapy CT scan) were often missing.

The application of radiomics to post-chemotherapy scans in NSGCTs can be used to predict the nature of residual masses before surgery (benign/malignant) to avoid overtreatment in a young population. However, these results remain insufficient for better selection of patients for surgery.

The association with validated clinical factors [20,21] or post-chemotherapy miRNA concentration (miR-371a-3p) [41] provides new opportunities to improve the discriminatory capacity of radiomics. 

Regarding radiomics, several studies have shown interest in harmonizing image acquisition, reconstruction, and delineation methods [25,33]. Even if some authors are currently trying to develop criteria for good practice in this field, in order to promote the reproducibility of data, there has been little consensus until now. This will likely be necessary to allow the emergence and reliability of this new tool.

## Figures and Tables

**Table 1:** Patient and tumor characteristics, overall and according to the persistence of malignant lesion in the surgical examination.

Characteristics	No Malignant Lesion		At Least One Malignant Lesion (N = 47)		Total		*p*-Value
	(N = 29)				(N = 76)		
**Age at diagnosis (years)**	N = 29		N = 47		N = 76		0.051 ^(1)^
Median-(Range)	26	(17.0; 45.0)	31	(17.0; 46.0)	28	(17.0; 46.0)	
Mean-SD	27.3	7	30.1	7.4	29.1	7.4	
**Initial pathology of the tumor**							0.02 ^(2)^
**Presence of teratoma**	4	13.80%	18	38.30%	22	28.90%	
Predominantly teratoma	2	6.90%	8	17%	10	13.20%	
Mixed with teratoma	2	6.90%	10	21.30%	12	15.80%	
**Absence of teratoma**	25	86.20%	29	61.70%	54	71.10%	
Predominantly embryonal carcinoma	17	58.60%	25	53.20%	42	55.30%	
Choriocarcinoma	1	3.40%	0		1	1.30%	
Predominantly vitelline tumor	2	6.90%	1	2.10%	3	3.90%	
Predominantly seminoma	3	10.30%	0		3	3.90%	
Burn-out tumor	0		2	4.30%	2	2.60%	
Mixed without teratoma	2	6.90%	1	2.10%	3	3.90%	
**Chemotherapy**							
≥3 cycles of platinum-based chemotherapy	29	100%	47	100%	76	100%	
**Time between end of chemotherapy and post-chemotherapy scan (weeks) (MD = 8)**	N = 24		N = 44		N = 68		0.95 ^(1)^
Median-(Range)	4.6	(1.3; 18.4)	4.4	(0.3; 364.9 *)	4.5	(0.3; 364.9)	
Mean-SD	5.6	3.8	14.5	54.6	11.4	44	
**Time between post-chemotherapy scan and surgery (weeks)**	N = 29		N = 47		N = 76		0.72 ^(1)^
Median-(Range)	6.9	(0.7; 27.9)	6.7	(0.0; 39.9)	6.7	(0.0; 39.9)	
Mean-SD	7.2	5.5	7.9	6.5	7.6	6.1	
**Number of persistent lesions**							0.80 ^(3)^
1	19	65.50%	26	55.30%	45	59.20%	
2 to 5	9	31.00%	19	40.40%	28	36.80%	
6 to 10	1	3.40%	1	2.10%	2	2.60%	
>10	0	0.00%	1	2.10%	1	1.30%	

^(1)^ Wilcoxon Mann Whitney test. ^(2)^ Chi square test. ^(3)^ Fisher’s exact test. MD = Missing data. * 1 patient lost to follow-up after chemotherapy.

**Table 2:** Pathological characteristics of the residual masses.

Anatomopathology of Residual Masses	Train (N = 99)		Test (N = 50)		Total (N = 149)	
**Malignant lesion**						
No (Necrosis or fibrosis)	34	34.3%	18	36.0%	52	34.9%
Yes	65	65.7%	32	64.0%	97	65.1%
- Viable tumor	10	10.1%	2	4.0%	12	8.1%
- Teratoma	55	55.6%	30	60.0%	85	57.0%

**Table 3:** Performance of the final model with selected thresholds on the training dataset and on the test dataset.

	Training Dataset				Test Dataset			
	AUC = 0.856 (0.772–0.939)				AUC = 0.823 (0.693–0.953)			
Threshold	Se (95% CI)	Sp (95% CI)	PPV (95% CI)	NPV (95% CI)	Se (95% CI)	Sp (95% CI)	PPV (95% CI)	NPV (95% CI)
(1) 0.5621	89.2% (79.1–95.6)	67.6% (49.5–82.6)	84.1% (73.3–91.8)	76.7% (57.7–90.1)	90.6% (75.0–98.0)	61.1% (35.7–82.7)	80.6% (64.0–91.8)	78.6% (49.2–95.3)
(2) 0.5966 (Youden)	86.2% (75.3–93.5)	76.5% (58.8–89.3)	87.5% (76.8–94.4)	74.3% (56.7–87.5)	84.4% (67.2–94.7)	66.7% (41.0–86.7)	81.8% (64.5–93.0)	70.6% (44.0–89.7)
(3) 0.5095	92.3% (83.0–97.5)	64.7% (46.5–80.3)	83.3% (72.7–91.1)	81.5% (61.9–93.7)	93.8% (79.2–99.2)	44.4% (21.5–69.2)	75.0% (58.8–87.3)	80.0% (44.4–97.5)

Threshold (1) = 0.5621 is the threshold maximizing the sensitivity while ensuring a specificity greater than 65%. Threshold (2) = 0.5966 is the threshold maximizing the Youden index, with equal weight to sensitivity and specificity. Threshold (3) = 0.5095 is the threshold maximizing the weighted sum (2 × Sensitivity + 1 × Specificity). Se: Sensitivity; Sp: Specificity; PPV: Positive Predictive Value; NPV: Negative Predictive Value; CI: Confidence Interval.

**Figure A1:**
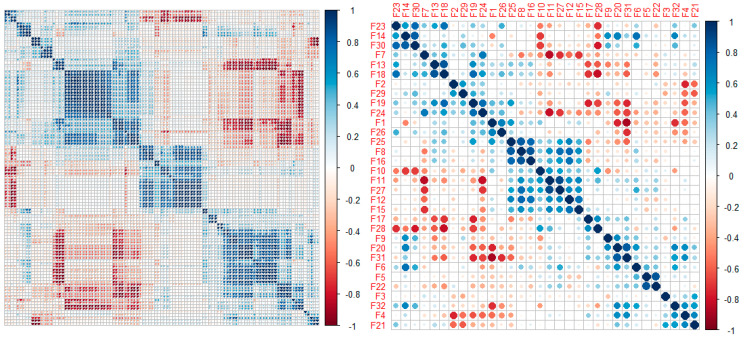
Correlation matrix of the 105 radiomics features and of the 32 radiomics features. (**Left panel**): considering the 105 radiomics features selected at the previous step. (**Right panel**): considering the 32 radiomics features resulting from this selection step.

## Data Availability

The dataset used and analysed during the current study are available from the corresponding author on reasonable request.

## Appendix A

**Table A1:** Clinical patient characteristics in the train dataset and in the test dataset.

Characteristics	Test (N = 58)		Train (N = 35)		Total (N = 93)	
**Age at diagnosis**	N = 58		N = 35		N = 93	
Median-(Range)	28.5	(17.0; 46.0)	28.0	(17.0; 44.0)	28.0	(17.0; 46.0)
Mean-SD	29.5	7.5	29.2	7.3	29.4	7.4
**Initial anatomopathology of the tumor**						
Presence of teratoma	19	32.8%	8	22.9%	27	29.0%
Absence of teratoma	39	67.2%	27	77.1%	66	71.0%
**Chemotherapy**						
≥3 cycles of platinum-based chemotherapy	58	100%	35	100%	93	100%
**Time between end of chemotherapy and post- chemotherapy scan (MD = 9)**	N = 54		N = 30		N = 84	
Median-(Range)	4.6	(0.3; 47.3)	4.7	(1.3; 364.9)	4.6	(0.3; 364.9)
Mean-SD	6.3	7.6	18.1	65.8	10.5	39.7
**Time between post-chemotherapy scan and surgery (in weeks)**	N = 58		N = 35		N = 93	
Median-(Range)	6.7	(0.0; 27.9)	6.0	(1.0; 39.9)	6.7	(0.0; 39.9)
Mean-SD	7.6	5.1	7.6	7.0	7.6	5.9
**Number of persistent lesions**						
1	29	50.0%	16	45.7%	45	48.4%
2 à 5	26	44.8%	16	45.7%	42	45.2%
6 à 10	2	3.4%	2	5.7%	4	4.3%
>10	1	1.7%	1	2.9%	2	2.2%

MD = Missing data.

As we split the dataset of lesions between the training and the test datasets, a patient may have lesions included in both datasets.

## Appendix B. Analysis of Correlation between Radiomics Features

This analysis included the 105 features selected from the previous step.

It was performed on the training dataset.

## Appendix C. Equation of the Resulting Elastic-Net Logistic Model

$P\left(\mathrm{residual}\;\mathrm{mass}\;\mathrm{is}\;\mathrm{malignant}\right|X=x)=\frac{e^{f(x)}}{1+e^{f(x)}}$, with:

- ${X=(X}_{1},X_{2},X_{3},X_{4},X_{5},X_{6},X_{7},X_{8})$
- $\begin{array}{ll} f\left(X\right) & =0.7910568-0.4787364\times X_{1}+0.0041544\times X_{2}-0.0136558\times X_{3}+\\ & 0.2026077\times X_{4}+0.1076146\times X_{5}-0.0306858\times X_{6}-0.0081377\times X_{7}+\\ & 0.4502361\times X_{8} \end{array}$

$X_{1}=$ *MORPHOLOGICAL_Surface To Volume Ratio (IBSI:2PR5)*

$X_{2}=$ *INTENSITY-BASED_Quartile Coefficient Of Dispersion (IBSI:9S40)*

$X_{3}=$ *GLCM (Grey level co-occurrence matrix)_Joint Maximum (IBSI:GYBY)*

$X_{4}$ = *GLCM_Normalised Inverse Difference Moment (IBSI:1QCO)*

$X_{5}$ = *GLCM_Correlation (IBSI:NI2N)*

$X_{6}$ = *NGTDM (Neighbourhood grey tone difference matrix)_Strength (IBSI:1X9X)*

$X_{7}$ = *GLSZM (grey level size zone matrix )_ Normalized Grey Level Non Uniformity (IBSI:Y1RO)*

$X_{8}$ = *GLSZM_Zone Size Entropy (IBSI:GU8N)*
